# Association of Depression, Anxiety, Stress and Stress-Coping Strategies with Somatization and Number of Diseases According to Sex in the Mexican General Population

**DOI:** 10.3390/healthcare10061048

**Published:** 2022-06-05

**Authors:** Aniel Jessica Leticia Brambila-Tapia, Fabiola Macías-Espinoza, Yesica Arlae Reyes-Domínguez, María Luisa Ramírez-García, Aris Judit Miranda-Lavastida, Blanca Estela Ríos-González, Ana Miriam Saldaña-Cruz, Yussef Esparza-Guerrero, Francisco Fabián Mora-Moreno, Ingrid Patricia Dávalos-Rodríguez

**Affiliations:** 1 Departamento de Psicología Básica, Centro Universitario de Ciencias de la Salud (CUCS), Universidad de Guadalajara, Guadalajara 44340, Mexico; 2 Departamento de Psicología Aplicada, Centro Universitario de Ciencias de la Salud (CUCS), Universidad de Guadalajara, Guadalajara 44340, Mexico; 3 Maestría en Psicología de la Salud, Centro Universitario de Ciencias de la Salud (CUCS), Universidad de Guadalajara, Guadalajara 44340, Mexico; yesica.reyes2393@alumnos.udg.mx (Y.A.R.-D.); maria.ramirez4357@alumnos.udg.mx (M.L.R.-G.); 4 Centro de Estudios sobre el Aprendizaje y Desarrollo (CEAD), Departamento de Psicología Básica, Centro Universitario de Ciencias de la Salud (CUCS), Universidad de Guadalajara, Guadalajara 44340, Mexico; aris.miranda@academicos.udg.mx (A.J.M.-L.); fabian.mora@academicos.udg.mx (F.F.M.-M.); 5 Unidad Médico Familiar #92, Instituto Mexicano del Seguro Social (IMSS), Guadalajara 44340, Mexico; blanca2282@hotmail.com; 6 Departamento de Fisiología, Centro Universitario de Ciencias de la Salud (CUCS), Universidad de Guadalajara, Guadalajara 44340, Mexico; ana.saldanac@academicos.udg.mx; 7 Doctorado en Farmacología, Centro Universitario de Ciencias de la Salud (CUCS), Universidad de Guadalajara, Guadalajara 44340, Mexico; yuss.tkd16@gmail.com; 8 Centro de Investigación Biomédica de Occidente (CIBO), Instituto Mexicano del Seguro Social (IMSS), Guadalajara 44340, Mexico; ingriddavalos@hotmail.com

**Keywords:** somatization, number of diseases, coping strategies, sex

## Abstract

Somatization and number of diseases are interrelated variables, whose association with stress-coping strategies, according to sex, has not been investigated. Therefore, the aim of this study was to investigate such association in a sample of the Mexican general population. The general population was invited to answer an electronic questionnaire via the social networks—e-mail, WhatsApp and Facebook—by the research team. A sample of 1008 adults was obtained, of which 62.2% were women, in whom we detected higher levels of negative psychological variables, somatization and number of diseases and lower levels of sleep quality. Positive moderate correlations were found between depresion, anxiety and stress with somatization, on one hand, and with the number of diseases, on the other, and negative moderate correlations were found between sleep quality and the two dependent variables. As for the coping strategies, self-blame, behavioral disengagement, denial, self-distraction and substance use were positively correlated with somatization. Of these, self-blame, substance use, and self-distraction also showed a positive correlation with number of diseases in both sexes. Negative correlations were detected for active coping and the two dependent variables in men and for religion and planning with somatization in women. In conclusion, the coping strategies showed significant correlations with somatization and number of diseases in both sexes.

## 1. Introduction

Somatization, described as the “tendency to experience and communicate somatic stress and to seek medical help for it” [1], has been associated with a variety of positive and negative psychological factors, with different tendencies in women compared to men [2] and with more of these psychological and sociodemographic factors associated in women, in whom somatization is also present at higher levels in comparison with men [2,3,4]. In addition, we found that the number of diseases has been significantly associated with somatization in previous reports in both genders [2,5].

The number of diseases is also increased in women when compared with men [2,6], which suggests that the increased number of negative psychological factors in women [7,8] could be related to the higher frequencies of somatization and number of diseases in this sex [2]. However, among the factors studied in association with somatization and number of diseases, no reports were found in relation to stress-coping strategies, which are defined as an individual’s attempts to use cognitive and behavioral strategies to manage and regulate pressures, demands and emotions in response to stress [9].

In a previous report, we showed that anxiety, depression and sleep quality were factors related to somatization in both sexes. Therefore, the aim of this study was to determine the association between coping strategies and both somatization and number of diseases, considering the previously associated variables of anxiety, depression and sleep quality. The study was performed in order to detect the coping strategies that are positively or negatively correlated with these two factors in each sex, which would permit the design of future preventing programs.

## 2. Subjects and Methods

An electronic questionnaire with sociodemographic and psychological instruments was sent via social networks, including WhatsApp, Facebook and e-mail, to the general population by the research team; this population included university students, relatives, friends, colleagues and acquaintances, many of whom re-sent the questionnaire.

The study was approved by the ethics and research committee of the Health Sciences University Center of the University of Guadalajara, and the participants gave their consent to participate in the questionnaire.

The socio-demographic data included sex, age, whether the participants had a romantic partner, schooling, whether they had children, whether they had a job, socioeconomic level (which represents the social and income level), monthly extra money (excluding necessary expenses), daily free hours and weekly physical activity hours, the presence of 21 different diseases (diabetes, hypertension, overweight, cancer, respiratory infections, gastrointestinal infections, allergies/asthma, gastritis/gastric ulcer, colitis/irritable colon, rheumatic diseases (rheumatoid arthritis, systemic lupus erythematosus, etc.), thyroid diseases, migraine, skin diseases (acne/neurodermatitis, etc.), sinusitis, kidney/bladder problems, anorexia/bulimia, hearth attack/angina pectoris, cerebral stroke, high cholesterol levels, anxiety and depression problems that require medication) and any additional disease in the last 6 months.

Sleep quality was measured with item 2 (which consists of 5 items) of the OVIEDO sleep questionnaire; these 5 questions were related to sleep problems and had 5 frequency options, from never to 6–7 days in a week [10]. Finally, smoking frequency and alcohol consumption frequency were measured with 6 options, i.e., from never to many times in a week.

The psychological measures included were: somatization, measured with the Patient Health Questionnaire 15 (PHQ-15), which evaluates 15 somatic symptoms with 3 frequency options for each symptom, i.e., not at all, bothered a little, bothered a lot [11]; stress, measured with the Cohen perceived stress scale (CPSS), which evaluates 14 phrases related to stress with 5 frequency options, i.e., from never to very frequent [12,13]; depression, measured with the CES-D scale, which consists of 10 questions related to depressive symptoms with 4 frequency options, i.e., from 0 days to 5–7 days in a week [14,15]; anxiety, measured with the GAD-7 scale, which consists of 7 phrases related to anxious symptoms with 4 frequency options, i.e., from never to almost all days [16,17]; and coping strategies, measured with the brief-COPE scale, which consists of 28 questions that evaluate 14 subscales (different coping strategies), each question having 4 frequency options, i.e., from “I never do it” to “I always do it” [18,19]. 

### Statistical Analysis

To describe the qualitative variables, we used frequencies and percentages, and for the quantitative ones, we used means and standard deviation. In order to compare the number of diseases and somatization between sexes, the Man–Whitney U test was used, considering the non-parametric distribution of the variables. Alpha Cronbach test was used to determine the reliability of each scale and sub-scale utilized. To perform comparisons between the psychological variables and sleep quality with the two dependent variables, we used the Spearman correlation test, considering the non-parametric distribution of the variables. In order to detect the distribution of the data, the Kolmogorov–Smirnov test was used. Finally, a multiple regression analysis, with the stepwise method, for both dependent variables by each sex was performed, in order to determine the variables most associated with somatization and number of diseases in men and women. All analyses were carried out with the software SPSS v. 25, and a *p* value < 0.05 was considered significant.

## 3. Results

We estimate that the questionnaire was sent to about 5000 persons through the different social networks, achieving a response rate of 20%. After excluding the questionnaires submitted by underage persons (24 persons), a total of 1008 participants were included, of which 62.2% were women. The socio-demographic data of the participants are reported in Table 1. Both sexes were comparable in age, schooling, whether they had children and socioeconomic level. Nevertheless, men showed significantly higher levels of daily free hours, weekly physical activity hours, smoking frequency and alcohol consumption frequency than women (*p* < 0.05).

The most frequent reported diseases in both sexes were anxiety, depression, skin problems, overweight, migraine and colitis/irritable colon, while the least frequent were cancer, cerebral stroke and heart attack (Table 2).

### 3.1. Bivariate Correlations between Psychological Variables and Both Somatization and Number of Diseases

The Cronbach alpha test for all the instruments used was above 0.7. In the case of the sub-scales of the brief COPE, most of them had scores above 0.6, with the exception of self-distraction, behavioral disengagement, denial and acceptance, which had scores above 0.5. In the case of the sub-scale venting, the Cronbach alpha was low, i.e., 0.35; therefore, we did not use this sub-scale in order to perform correlations and comparisons. 

When we compared the somatization and number of diseases between sexes, women reported significantly higher somatization, number of diseases, stress, depression and anxiety than men; likewise, women reported lower sleep quality than men (Table 3).

#### 3.1.1. Correlations with Stress, Depression, Anxiety and Sleep Quality

In the bivariate correlations, both sexes showed significant positive moderate correlations between stress, anxiety and depression with somatization, on one hand, and with the number of diseases, on the other, with higher correlations for depression in women and for anxiety in men. In addition, sleep quality showed significant negative moderate correlations with both dependent variables (Table 4).

When anxiety and depression problems were not included in the number of diseases, the correlations between the three psychological variables diminished, but were still significant for both sexes. The correlation of number of diseases with anxiety was as follow: for men, r = 0.380, *p* <0.01; for women, r = 0.313, *p* < 0.01. The same correlation with depression was, for men, r = 0.311, *p* < 0.01; for women, r = 0.306, *p* < 0.01. The correlation with stress was, for men, r = 0.290, *p* < 0.01; for women, r = 0.237, *p* < 0.01. The correlation with sleep quality was, for men, r = −0.262, *p* < 0.01; for women, r = −0.328, *p* < 0.01. When overweight was also excluded from the number of diseases, the correlations remained similar as those found when excluding anxiety and depression problems. Somatization and number of diseases showed significant positive moderate correlations between them in both sexes: men: r = 0.545, *p* < 0.001, and women: r = 0.547, *p* < 0.001.

#### 3.1.2. Correlations with Coping Strategies

In relation to coping strategies, women showed significant low positive correlations between somatization and self-blame, denial, behavioral disengagement, substance use, self-distraction and humor, and significant low negative correlations between somatization and planning and religion. For the number of diseases, women showed significant low positive correlations with self-blame, substance use, denial, behavioral disengagement, self-distraction, humor and instrumental support.

Men showed significant low positive correlations between somatization and self-blame, behavioral disengagement, denial, self-distraction and substance use. For the number of diseases, men showed significant low positive correlations with self-blame, substance use and self-distraction, and significant low negative correlations with humor and active coping.

#### 3.1.3. Bivariate Correlations between Socio-Demographic Variables and Somatization and Number of Diseases

##### Correlations with Somatization

Sociodemographic variables also showed low but significant negative correlations with somatization and number of diseases. In women, we found negative correlations between somatization and age (r = −0.256, *p* < 0.01), weekly physical activity hours (r = −0.214, *p* < 0.01), monthly extra money (r = −0.182, *p* < 0.01), whether they had children (r = −0.158, *p* < 0.01), schooling (r = −0.134, *p* < 0.01), socioeconomic level (r = −0.129, *p* < 0.01), daily free hours (r = −0.085, *p* < 0.05) and whether they worked (r = −0.080, *p* < 0.05), and a low positive correlation with smoking frequency (r = 0.093, *p* < 0.05). 

In men, we found negative correlations between somatization and monthly extra money (r = −0.164, *p* < 0.01), weekly physical activity hours (r = −0.149, *p* < 0.01) and whether they had children (r = −0.115, *p* < 0.01).

##### Correlations with Number of Diseases

For the number of diseases, in women, we found low but significant negative correlations with whether they had children (r = −0.143, *p* < 0.01), socioeconomic level (r = −0.112, *p* < 0.01) and weekly physical activity hours (r = −0.101, *p* < 0.01), and a low positive correlation with smoking frequency (r =0.110 *p* < 0.05). In men, we found low but significant negative correlations between the number of diseases and weekly physical activity hours (r = −0.132, *p* < 0.05), and a low positive correlation with age (r = 0.105, *p* < 0.05) and smoking frequency (r = 0.105, *p* < 0.05).

### 3.2. Multiple Regression Analysis for Somatization and Number of Diseases

In the multiple regression analysis for somatization in men and women, adjusting for the sociodemographic variables, the most associated variable was depression followed by number of diseases, sleep quality (negatively associated) and anxiety. In contrast, for number of diseases, the most associated variables were sleep quality (negatively associated), anxiety and schooling (Table 5 and Table 6).

## 4. Discussion

We found that somatization and number of diseases were associated and that they were also associated with the main negative psychological variables, i.e., anxiety, depression and stress. Likewise, they were negatively correlated with sleep quality, in both sexes. We also showed that typical maladaptive coping patterns were positively correlated with somatization and number of diseases (mainly self-blame) in both sexes and that active coping correlated negatively with somatization and number of diseases only in men. 

As previously shown, negative psychological variables, somatization and number of diseases reached higher levels in women than in men [2,3,4,5]. We also detected that sleep quality was lower in women than in men, which can be related to the increased frequency of the negative psychological variables in this sex, considering that sleep quality is negatively correlated with anxiety, stress and depression [2,7]. Interestingly, the most frequent diseases in both sexes were anxiety and depression problems that required medication (58% and 33% in women, and 38% and 26% in men), which were more frequent in women than in men. This is an unexpected finding, together with the relatively low frequency of diabetes and hypertension, which were reported as the most frequent diseases in Mexico (~10% for diabetes, and 30% for hypertension) [20].

These differences can be explained by considering that the population studied was mainly young and could present more psychological problems and less metabolic ones. 

With respect to the correlation analysis, we found that the variables most associated with somatization in both sexes were anxiety, depression and stress, with moderate positive correlations, as well as sleep quality, with moderate negative correlations. 

The coping strategies showed lower correlations with somatization in both sexes. It is of interest that self-blame showed the highest correlations in both sexes, followed by behavioral disengagement, denial, self-distraction and substance use; these correlations indicate a possible indirect correlation between these coping strategies and somatization, considering that these strategies were positively correlated with the negative psychological variables in both sexes (data not shown) and are typically considered as maladaptive coping strategies [21,22]. Additionally, women showed a low positive correlation between the coping strategy humor and both somatization and number of diseases, which suggests that this coping strategy may be maladaptive only in women. This is supported by the fact that humor also showed very low positive correlations with stress, depression and anxiety in women (data not shown).

The coping strategies negatively correlated with somatization were active coping in men, and religion and planning in women, which coincides with the fact that these strategies have been classified as adaptive [21,22]. However, the positive correlations were stronger than the negative correlations, which suggests that maladaptive coping strategies may contribute to somatization more than adaptive ones.

As previously shown, a positive moderate correlation was found between somatization and number of diseases in both sexes [2]. This suggests that somatization is also a consequence of disease presence. For the number of diseases, the three negative psychological variables showed moderate positive correlations, and sleep quality showed moderate negative correlations with this variable in both sexes. These results coincide with our previous report showing a significant positive correlation between anxiety and depression with number of diseases in women, and a negative correlation between sleep quality and number of diseases also in women [2]. However, in the case of men, we now detected a positive correlation between anxiety and depression and number of diseases, and a negative correlation between sleep quality and number of diseases that had not been previously identified, which can be explained by the increased sample size and the higher number of diseases considered in this study, which permitted us to better evaluate these associations. These correlations are in concordance with psychoneuroendocrinology findings, which correlate negative psychological variables with inflammation and oxidative stress that contribute to disease development [23,24]. In addition, the importance of sleep quality in order to maintain a good health condition, with no or less diseases, has been highlighted; however, bilateral relationships are also possible, and only longitudinal designs can clarify this. In this sense, a recent report showed that sleep promotes the activity of DNA damage response proteins in zebrafish [25], relating sleep with DNA repair, which could be a protective factor for disease development.

In addition, self-blame and substance use were the only coping strategies positively associated (low correlation) with number of diseases in both sexes; women also showed low positive correlations with behavioral disengagement, denial and humor, while men showed low negative correlations with humor and active coping. These results confirm the correlations found for somatization, although for this variable, a decreased number of associated coping strategies and a diminishment in the strength of the associations were observed. These changes were expected, considering that disease presence is a variable related to more causes than somatization, which, in turn, is more associated with psychological variables.

The multivariate analysis confirmed the results of the bivariate analysis, indicating that depression, number of diseases, sleep quality and anxiety were the variables that most explained the variability of somatization. Likewise, sleep quality and anxiety were the variables that most explained the number of diseases. These findings coincide with our previous report [2] where sleep quality was included in the multivariate analysis for the number of diseases in both sexes. However, differently from this previous report, we suggest that the negative psychological variables studied, i.e., anxiety, depression and stress, together with sleep quality, equally contribute to disease development in both sexes.

This study has the following limitations: the sample was predominantly young and was not randomly selected, which could decrease the representativeness of the Mexican population, restricting it to young and educated people, from a medium socioeconomic level, who have access to the internet and social networks, and not representing older people and those from lower socioeconomic levels. In addition, the cross-sectional design of the study did not permit us to demonstrate causality between the studied variables, being bilateral relationships plausible, mainly between sleep quality and somatization and number of diseases. Finally, the estimated response rate of the questionnaire was low (20%), which can represent a bias by considering that it could be answered by people who had more interest in the theme and/or who presented more emotional problems/concerns. 

In conclusion, somatization and number of diseases showed a higher frequency in women than in men. These variables were mainly related (positively) with the negative psychological variables of depression, anxiety and stress, as well as with sleep quality (negatively) in both sexes. Likewise, these variables showed lower but significant positive correlations with some maladaptive strategies, mainly, self-blame, in both sexes. Additionally, some of these strategies, considered as adaptive, showed negative correlations with somatization and number of diseases, being more constant in the case of men, where active coping was negatively correlated with these two variables. Humor showed different correlations in men and women, suggesting that this strategy is adaptive in men but maladaptive in women. Further studies with larger sample sizes, longitudinal designs and performed in different populations should be performed to confirm these results.

## Figures and Tables

**Table 1:** Sociodemographic variables in the studied population.

Variable	Women, *n* = 627	Men, *n* = 381
Age, mean ± SD	29.89 ± 11.08	30.13 ± 11.36
With romantic partner, *n* (%)	382 (60.90)	205 (53.80)
With children, *n* (%)	192 (30.60)	102 (26.80)
With job, *n* (%)	376 (60.00)	259 (68.00)
Educational level - Elementary school - High school - Preparatory - Bachelor’s degree - Technical career - Master’s degree - Ph.D. degree	2 (0.30) 10 (1.60) 136 (21.70) 352 (56.10) 30 (4.80) 74 (11.80) 23 (3.70)	0 (0.0) 7 (1.84) 92 (24.14) 205 (53.81) 21 (5.51) 36 (9.45) 20 (5.25)
Socioeconomic level - Very low - Low - Medium - High	0 (0.00) 106 (16.90) 501 (79.90) 20 (3.20)	4 (1.10) 61 (16.00) 308 (80.80) 8 (2.10)
Daily free hours, mean ± SD	4.08 ± 2.68	4.53 ± 2.80
Weekly physical activity hours, median (range)	2 (0−20)	3 (0−35)
Smoking frequency, mean ± SD	1.63 ± 1.42	1.97 ± 1.74
Alcohol consumption, mean ± SD	2.70 ± 1.40	3.07 ± 1.55

SD: Standard deviation. Smoking and alcohol consumption were measured from 1, never, to 6, many times, in a week.

**Table 2:** Frequency of the self-reported diseases in each sex.

Disease, *n* (%)	Women, *n* = 627	Men, *n* = 381
Anxiety problems	364 (58.05)	144 (37.80)
Depression problems	204 (32.54)	98 (25.72)
Skin diseases	213 (33.97)	60 (15.75)
Overweight	176 (28.07)	93 (24.41)
Migraine	190 (30.30)	59 (15.49)
Colitis/Irritable colon	192 (30.62)	48 (12.60)
Gastritis/gastric ulcer	145 (23.13)	51 (13.39)
Allergies/asthma	99 (15.79)	36 (9.45)
Gastrointestinal infections	75 (11.96)	45 (11.81)
Respiratory infections	65 (10.37)	28 (7.35)
Sinusitis	34 (5.42)	13 (3.41)
Thyroid problems	30 (4.78)	5 (1.31)
Hypertension	19 (3.03)	27 (7.09)
Anorexia/bulimia	27 (4.30)	2 (0.52)
High cholesterol	21 (3.35)	15 (3.94)
kidney/bladder problems	17 (2.71)	8 (2.10)
Diabetes	16 (2.56)	8 (2.10)
Rheumatic diseases	15 (2.39)	6 (1.57)
hearth attack/angina pectoris	1 (0.16)	2 (0.52)
Cerebral stroke	0 (0.00)	0 (0.00)
Cancer	0 (0.00)	0 (0.00)

**Table 3:** Comparison of psychological variables and number of diseases between sexes.

Variable	Men (*n* = 381) Mean ± SD	Women (*n* = 627) Mean ± SD	*p* Value
Somatization	1.47 ± 0.30	1.75 ±0.36	<0.001
Number of diseases	2.02 ± 1.80	3.11 ± 2.18	<0.001
Sleep quality	3.75 ± 0.89	3.55 ± 0.93	0.001
Stress	2.71 ± 0.68	3.03 ± 0.65	< 0.001
Depression	1.01 ± 0.61	1.27 ± 0.64	<0.001
Anxiety	0.99 ± 0.76	1.35 ± 0.81	<0.001

SD: Standard deviation, somatization scale (PHQ-15), range: 1–3, Number of diseases, range: 0–22, sleep quality (OVIEDO scale) range: 1–5, stress scale (CPSS) range of 1–5; depression scale (CES-D) range: 0–3 and anxiety scale (GAD-7) range: 0–3.

**Table 4:** Bivariate correlations between psychological variables and coping strategies and somatization and number of diseases in each sex.

	Men, *n* = 381		Women, *n* = 627	
Variable	Somatization	Number of Diseases	Somatization	Number of Diseases
Depression	0.566 **	0.423 **	0.631 **	0.449 **
Anxiety	0.604 **	0.462 **	0.622 **	0.444 **
Stress	0.496 **	0.383 **	0.525 **	0.365 **
Sleep quality	−0.571 **	−0.348 **	−0.589 **	−0.430 **
Coping strategies				
Religion	−0.090	−0.035	−0.101 *	−0.044
Substance use	0.108 *	0.122 *	0.212 **	0.188 **
Self-blame	0.353 **	0.204 **	0.340 **	0.278 **
Behavioral disengagement	0.193 **	0.098	0.216 **	0.106 **
Emotional support	0.019	−0.038	0.018	0.055
Instrumental support	0.045	−0.070	0.057	0.079
Active coping	−0.112 *	−0.114 *	−0.075	0.007
Planning	−0.036	0.073	−0.106 **	−0.010
Self-distraction	0.167 **	0.105 *	0.197 **	0.098 *
Denial	0.168 **	0.019	0.267 **	0.146 **
Positive reframing	−0.042	−0.089	−0.045	−0.002
Acceptance	−0.061	−0.087	−0.062	−0.003
Humor	0.047	−0.115 *	0.156 **	0.092 *

* *p* < 0.05, ** *p* < 0.01. *p* value obtained with Spearman correlation test.

**Table 5:** Multivariate regression analysis for somatization.

	Men					
Variable	Not Standardized Coefficient Β	Coefficient Β CI 95% (Lower and Upper Limits)		Standardized Beta Coefficient	*p* Value	Change in R^2^
Constant	1.372	1.214	1.528	-	0.000	-
Depression	0.064	0.002	0.126	0.126	0.043	0.349
Number of diseases	0.059	0.045	0.073	0.348	0.000	0.115
Sleep quality	−0.066	−0.097	−0.036	−0.195	0.000	0.039
Anxiety	0.091	0.045	0.138	0.225	0.000	0.016
Denial	0.052	0.016	0.089	0.106	0.005	0.009
Humor	0.032	0.009	0.056	0.100	0.007	0.005
	**Women**					
Constant	1.826	1.675	1.977	-	0.000	-
Depression	0.109	0.058	0.159	0.195	0.000	0.395
Number of diseases	0.038	0.028	0.048	0.232	0.000	0.079
Sleep quality	−0.091	−0.118	−0.065	−0.238	0.000	0.043
Anxiety	0.091	0.053	0.129	0.208	0.000	0.022
Monthly extra money	−0.020	−0.038	0.002	−0.063	0.029	0.006
Age	−0.002	−0.004	−0.0003	−0.070	0.019	0.004
Weekly physical activity hours	−0.007	−0.014	−0.001	−0.065	0.022	0.004

The unstandardized coefficient Β represents the direct contribution of each variable to the dependent variable, while the standardized beta coefficient is obtained by converting the direct contributions to typical contributions (standard deviations of the dependent variable), which permits to determine the relative value of each variable in relation to the dependent variable. R of the model for men: 0.729, R^2^ = 0.532. R of the model for women: 0.743, R^2^ = 0.553.

**Table 6:** Multivariate regression analysis for number of diseases.

	Men					
Variable	Not Standardized Coefficient Β	Coefficient Β CI 95% (Lower and Upper Limits)		Standardized Beta Coefficient	*p* Value	Change in R^2^
Constant	1.674	0.468	2.880	-	0.007	-
Sleep quality	−0.389	−0.596	−0.182	−0.194	0.000	0.192
Anxiety	0.861	0.612	1.110	0.360	0.000	0.029
Schooling	0.253	0.103	0.403	0.151	0.001	0.022
Instrumental support	−0.274	−0.497	−0.050	−0.111	0.017	0.012
	**Women**					
Constant	2.815	1.552	4.077	-	0.000	-
Sleep quality	−0.621	−0.821	−0.421	−0.264	0.000	0.196
Anxiety	0.477	0.188	0.767	0.178	0.001	0.054
Schooling	0.225	0.082	0.369	0.110	0.002	0.010
Depression	0.490	0.098	0.881	0.143	0.014	0.009
Substance use	0.261	0.025	0.498	0.079	0.030	0.006

The unstandardized coefficient Β represents the direct contribution of each variable to the dependent variable, while the standardized beta coefficient is obtained when the direct contributions are converted to typical contributions (standard deviations of the dependent variable), which permits to determine the relative value of each variable in relation to the dependent variable. R of the model for men: 0.505, R^2^ = 0.255. R of the model for women: 0.524, R^2^ = 0.275. * Somatization was not included in these analyses, considering that this variable, rather than a cause, is a consequence of the number of diseases.

## Data Availability

Data that support the findings of the study are available upon reasonable request.

## References

[1] Lipowski Z.J. (1988). Somatization: The concept and its clinical application. Am. J. Psychiatry.

[2] Brambila-Tapia A.J.L., Saldaña-Cruz A.M., Meléndez-Monreal K.C., Esparza-Guerrero Y., Martínez-Hernández A., Rosales-Torres B.G., Ríos-González B.E. (2021). Association of personal, behavioral and positive psychological variables with somatization and number of diseases in Mexican general population: The influence of gender. Psychol. Health Med..

[3] Ladwig K.H., Marten-Mittag B., Erazo N., Gündel H. (2001). Identifying Somatization Disorder in a Population-Based Health Examination Survey: Psychosocial Burden and Gender Differences. Psychosomatics.

[4] Vargas-Prada S., Coggon D., Ntani G., Walker-Bone K., Palmer K.T., Felli V.E., Harari R., Barrero L.H., Felknor S.A., Gimeno D. (2016). Descriptive Epidemiology of Somatising Tendency: Findings from the CUPID Study. PLoS ONE.

[5] Brambila-Tapia A.J.L., Meda-Lara R.M., Palomera-Chávez A., de-Santos-Ávila F., Hernández-Rivas M.I., Bórquez-Hernández P., Juárez-Rodríguez P. (2019). Association between personal, medical and positive psychological variables with somatization in university health sciences students. Psychol. Health Mes..

[6] Case A., Paxson C. (2005). Sex differences in morbidity and mortality. Demography.

[7] Schlarb A.A., Claßen M., Hellmann S.M., Vögele C., Gulewitsch M.D. (2017). Sleep and somatic complaints in university students. J. Pain Res..

[8] Garaigordobil M., Perez J.I., Mozaz M. (2008). Self concept, self-esteem and psychopathological symptoms. Psicothema.

[9] Lazarus R., McHugh S., Vallis T.M. (1986). Coping Strategies. Illness Behavior: A Multidisciplinary Model.

[10] Bobes-García J., González G., Portilla P., Sáiz-Martínez D.A., Bascarán-Fdez M., Iglesias-Álvarez G., Fdez-Dominguez J.M. (2000). Propiedades psicométricas del cuestionario de Oviedo de sueño. Psicothema.

[11] Ros-Montalbán S., Comas Vives A., Garcia-Garcia M. (2010). Validation of the Spanish version of the PHQ-15 questionnaire for the evaluation of physical symptoms in patients with depression and/or anxiety disorders: DEPRE-SOMA study. Actas Esp. Psyquiatr..

[12] Cohen S., Kamarck T., Mermelstein R. (1983). A global measure of perceived stress. J. Health Soc. Behav..

[13] Remor E. (2006). Psychometric properties of a European Spanish version of the perceived Stress Scale (PSS). Span. J. Psychol..

[14] Radloff L. (1977). The CES-D Scale: A self-report depression scale for research in the general population. Appl. Psychol. Meas..

[15] González-Forteza C., Jiménez-Tapia J.A., Ramos-Lira L., Wagner F.A. (2008). Aplicación de la Escala de Depresión del Center of Epidemiological Studies en adolescentes de la Ciudad de México. Salud Publica Mex..

[16] Spitzer R.L., Kroenke K., Williams J.B.W., Löwe B. (2006). A Brief Measure for Assessing Generalized Anxiety Disorder. Arch. Intern. Med..

[17] Garcia-Campayo J., Zamorano E., Ruiz M.A., Pardo A., Perez-Paramo M., Lopez-Gomez V., Freire O., Rejas J. (2010). Cultural adaptation into Spanish of the generalized anxiety disorder-7 (GAD-7) scale as a screening tool. Health Qual. Life Outcomes.

[18] Carver C.S. (1997). You want to measure coping but your protocol’ too long: Consider the brief cope. Int. J. Behav. Med..

[19] Morán C., Landero R., González M.T. (2010). COPE-28: Un análisis psicométrico de la versión en español del BriefCOPE. Univ. Psychol..

[20] Tapia-Conyer R., Gallardo-Rincón H., Saucedo-Martinez R. (2013). CASALUD: An innovative health-care system to control and prevent non-communicable diseases in Mexico. Perspect. Public Health.

[21] Cooper C., Katona C., Orrell M., Livingston G. (2006). Coping strategies and anxiety in caregivers of people with Alzheimer’s disease: The LASER-AD study. J. Affect. Disord..

[22] Meyer B. (2006). Coping with severe mental illness: Relations of the brief COPE with symptoms, functioning, and well-being. J. Psychopathol. Behav. Assess..

[23] Furman D., Campisi J., Verdin E., Carrera-Bastos P., Targ S., Franceschi C., Ferrucci L., Gilroy D.W., Fasano A., Miller G.W. (2019). Chronic inflammation in the etiology of disease across the life span. Nat. Med..

[24] Liguori I., Russo G., Curcio F., Bulli G., Aran L., Della-Morte D., Gargiulo G., Testa G., Cacciatore F., Bonaduce D. (2018). Oxidative stress, aging, and diseases. Clin. Interv. Aging.

[25] Zada D., Sela Y., Matosevich N., Monsonego A., Lerer-Goldshtein T., Nir Y., Appelbaum L. (2021). Parp1 promotes sleep, which enhances DNA repair in neurons. Mol. Cell.

